# Feasibility analysis of Sinomenine alleviating fibrosis of filtering bleb after glaucoma filtering surgery: a mini review

**DOI:** 10.3389/fmed.2025.1607500

**Published:** 2025-07-08

**Authors:** Xin Xun, Xiyuan Liu, Pai Zhou, Chengliang Wu, Qinghua Peng

**Affiliations:** ^1^Hunan University of Chinese Medicine, Changsha, China; ^2^Key Laboratory of Traditional Chinese Medicine for Prevention and Treatment of Eye, Ear, Nose and Throat Diseases, Changsha, China; ^3^First Affiliated Hospital of Hunan University of Chinese Medicine, Changsha, China

**Keywords:** Sinomenine, glaucoma, glaucoma filtering surgery, filtering bleb, fibrosis

## Abstract

Fibrosis of the filtering bleb remains the predominant cause of glaucoma filtering surgery failure, mediated by interconnected pathological processes including postoperative local inflammation, aberrant fibroblast proliferation, and deposition of the extracellular matrix (ECM). The antimetabolite drugs 5-fluorouracil (5-FU) and mitomycin C (MMC) are effective in preventing filtering bleb fibrosis, but their non-specific cytotoxic effects necessitate the development of targeted therapeutic alternatives. Fibrosis is a group of diseases with similar pathological mechanisms and molecular features. By analyzing evidence of Sinomenine's (SIN) anti-fibrotic effects across multiple organs, this study explores its potential use in glaucoma filtration surgery (GFS) to reduce scarring: (1) SIN inhibits trauma-induced NF-κB activation in Tenon's fibroblasts (TFs), reduces neutrophil and macrophage infiltration, and suppresses cytokine cascades. Besides, SIN targets the phosphatidylinositol-3-kinase (PI3K)/Akt pathway to attenuate macrophage M2 polarization and neutrophil recruitment, thereby interrupting fibrotic progression. (2) SIN suppresses transforming growth factor-β (TGF-β)/Smad3 signaling and inhibits the transdifferentiation of fibroblasts into α smooth muscle actin (α SMA) expressing myofibroblasts (MFs). SIN also blocks fibroblast proliferation and migration via PI3K/Akt/mTORC1 axis inhibition, restraining myofibroblast differentiation—the central pathological event in filtering bleb scarring. SIN shows antifibrotic efficacy, and feasibility studies on its application may offer novel insights into antifibrotic strategies.

## Graphical Abstract

This graphical abstract systematically delineates the formation mechanisms of filtering bleb fibrosis, current therapeutic strategies, and the potential role of SIN in inhibiting fibrotic progression. The development of filtering bleb fibrosis is closely associated with inflammatory responses after GFS, TFs activation, and their transdifferentiation into myofibroblasts. Current clinical interventionsprimarily employ glucocorticoids, 5-FU and MMC. SIN, a bioactive alkaloid, exhibits anti-inflammatory, fibroblast proliferation-inhibiting, and apoptosis-promoting properties, and may serve as a potential therapeutic approach for suppressing filtering bleb fibrosis. 
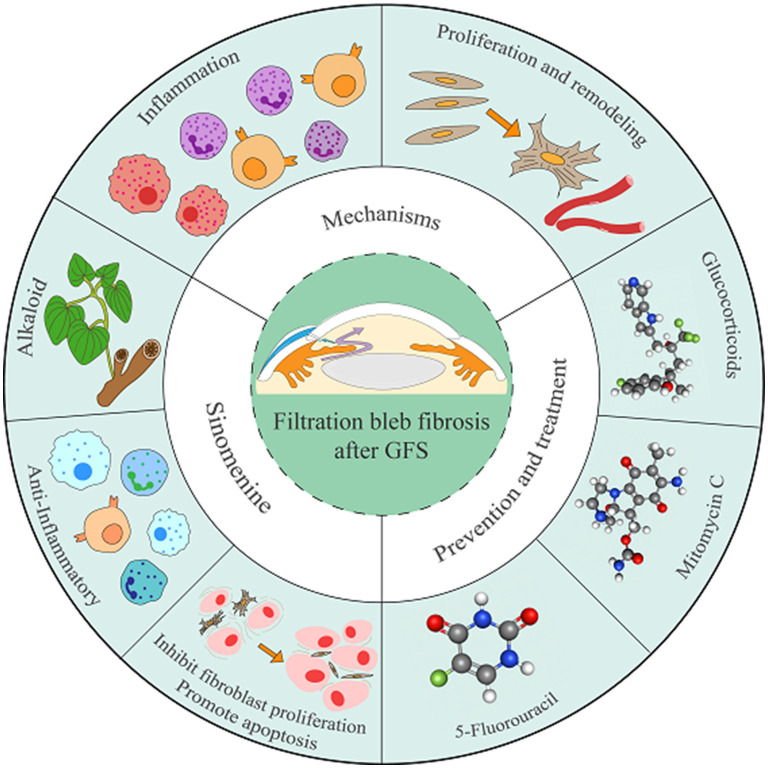

## 1 Introduction

Glaucoma is a group of optic neuropathies characterized by progressive degeneration of retinal ganglion cells, leading to optic nerve damage and visual field loss. Primary open-angle glaucoma (POAG) is the most common type of glaucoma worldwide. Most cases of POAG may progress to total blindness without the patient experiencing any pain or discomfort. Glaucoma has become the leading cause of irreversible blindness worldwide (1–3). It is projected that by 2040, the number of glaucoma cases will reach 110 million worldwide, with up to 60% of the cases in Asia, posing a serious threat to global visual health and quality of life (4, 5). Multiple factors influence the risk of developing glaucoma, including age, race, family history, corneal thickness, systemic hypotension, cerebrospinal fluid pressure, intraocular pressure (IOP), and vascular disorders (6). Pathological elevation of intraocular pressure (IOP) is an independent risk factor for glaucoma. Lowering IOP through various treatments is an established strategy for preventing vision loss in patients with glaucoma (7). When medication and laser therapies fail to adequately control IOP, surgical intervention becomes the primary therapeutic consideration. Glaucoma filtration surgery (GFS) is the first-line surgical option in current clinical practice. This surgical procedure facilitates aqueous humor drainage through the creation of a subconjunctival filtration channel to regulate IOP. The key determinant of this procedure is the establishment and maintenance of a patent and functioning filtering bleb. However, the procedure damages the ocular tissues, activating the wound repair cascade. Inflammatory response during the early healing phase may drive fibrosis. This process involves the proliferation and differentiation of fibroblasts and the excessive deposition of extracellular matrix (ECM). These changes cause postoperative fibrosis of the filtering bleb, which in turn damages the function of the filtering bleb, block the outflow of aqueous humor, and ultimately lead to failure of GFS (8, 9). Therefore, the prevention of excessive fibrosis of the surgical area has been a major research focus in GFS.

Antimetabolite agents such as 5-fluorouracil (5-FU) and mitomycin C (MMC) are standard therapeutic agents to prevent fibrosis of filtering blebs. Although these agents effectively prolong the survival duration of filtering blebs and enhance the long-term success rate of glaucoma filtration surgery (GFS), they carry significant risks of ocular complications. These include thin-walled cystic blebs predisposing to late leakage, heightened infection risks, chronic hypotony-associated pathologies, and corneal epithelial damage (10, 11). Investigating alternative therapeutic approaches could help improve clinical outcomes.

Sinomenine (SIN) is an alkaloid monomer derived from *Sinomenium acutum*, a plant of the Menispermaceae family. Its molecular formula is C_19_H_23_NO_4_, and its molecular weight is 329.39 (12). SIN shows immunosuppressive, anti-inflammatory, apoptosis-inducing, antihypertensive, analgesic, and other pharmacological effects, and it is potentially efficacious for antifibrotic applications. SIN exerts its antifibrotic effects primarily through inhibiting signaling pathways [including transforming growth factor-β (TGF-β)/Smad, phosphatidylinositol-3-kinase (PI3K)/Akt, and NF-κB], suppressing inflammatory cytokine release, and downregulating fibroblast activation. Fibrosis in the filtering bleb shares a similar pathology with fibrosis of other tissues and organs, but there have been no reports of SIN inhibiting fibrosis in the filtering bleb. In this review, we analyze the mechanisms of fibrosis of the filtering bleb and the pharmacological effects of SIN in order to evaluate the feasibility of using SIN to inhibit fibrosis after GFS.

## 2 Mechanism of fibrosis of filtering bleb after GFS

### 2.1 Healing process

The healing process of the filtering bleb after GFS is in accordance with general wound healing, following the pattern of hemostasis, inflammation, cellular proliferation, and tissue remodeling (13, 14). After the procedure, platelets aggregate at damaged vessels and form fibrin clots, with platelets releasing inflammatory factors, growth factors, and activating the inflammatory response. During the inflammatory phase, cellular infiltration is increased. The cytokine secretion drives fibroblast activation, and fibroblasts sustainably proliferate and transdifferentiate into myofibroblasts (MFs) which express α-smooth muscle actin (α-SMA) (15). Large amounts of ECM are released by MFs and promote protein deposition. This causes migration and epithelialization of epithelial cells, neovascularization, and granulation tissue formation, leading to tissue remodeling and eventual scar formation (16).

### 2.2 Injury-induced activation

The Tenon's capsule, also known as the fascial sheath of the eyeball, is a dense connective tissue that wraps around the outer sclera and is populated by fibroblasts (17). Fibroblasts are able to transdifferentiate into MFs, which play a central role in ECM synthesis and secretion. They facilitate wound healing, and are also involved in numerous fibrotic diseases. Fibrosis of the filtering bleb is mainly induced by the proliferation, migration and contraction of Tenon's fibroblasts (TFs). Hyperproliferation and differentiation of TFs are important for postoperative fibrosis in the filtering area. Surgical procedures inevitably cause some degree of damage to the cornea, conjunctiva, Tenon's capsule, and sclera. During the healing process of the filtering area, excessive fibrosis develops. Studies have shown (18) that patients with glaucoma exhibit significant fibrotic changes in TFs, as evidenced by the transdifferentiation of fibroblasts to myofibroblasts, and associated changes such as mitochondrial fission, ECM remodeling, proliferation, inflammation, and apoptosis. These changes may be related to their pathogenesis and/or the damage caused by local treatment.

### 2.3 Inflammatory response

Inflammation is one of the crucial factors in the formation of scarring and occurs in the early stages of wound healing. Excessive and prolonged inflammation impairs wound healing and promotes scar formation (19). The acute inflammatory response is characterized by increased exudation, thickening of the filtering bleb, dense collagenous tissue, and hyperproliferation of fibroblasts, and excessive angiogenesis. As a result of the activation of the endogenous coagulation cascade reaction, large amounts of cytokines and growth factors are released, causing the wound healing phase to prolong the inflammatory phase. These factors include tumor necrosis factor-α (TNF-α), interleukin (IL), transforming growth factor-β (TGF-β), platelet-derived growth factor (PDGF), and vascular endothelial growth factor (VEGF), which are able to recruit and activate fibroblasts and vascular endothelial cells in turn (20–22). TNF-α, as an inflammatory mediator, promotes inflammatory cell recruitment in subconjunctival tissues and exerts a destructive effect on trabecular cells by up-regulating the expression of pro-inflammatory factors, such as IL-1 and IL-6. Fibroblast proliferation exhibits a higher rate and longer duration after TNF-α intervention (22). IL-6 plays a crucial role in the development of scar formation after GFS by accelerating the fibrotic process by promoting the proliferation of CD4+ T cells, inhibiting autophagy, enhancing endoplasmic reticulum stress, and promoting the transformation of fibroblasts in the early stages of inflammation (23, 24). TGF-β in atrial fluid has been shown to promote fibroblast migration, proliferation, and differentiation, as well as increase the expression of type I collagen and fibronectin, leading to scarring of postoperative filtration channels (25, 26). VEGF directly modulates a spectrum of pro-fibrotic genetic pathways by orchestrating myofibroblast differentiation through upregulation of collagen synthesis and α-smooth muscle actin (α-SMA) expression, thereby mediating fibrotic progression (27). PDGF induces vascular repair, promotes proliferation and migration of macrophages and fibroblasts to the wound site, and stimulates fibroblasts to transdifferentiate into myofibroblasts, which enhances the ECM and angiogenesis and promotes scarring (28, 29).

### 2.4 Fibroblast transdifferentiation

TFs are activated by a combination of cytokines and growth factors, start to proliferate and migrate, and undergo sustained transdifferentiation into MFs. MFs synthesize large amounts of collagen-rich ECM. The disordered collagen fibers induce abnormal cell movement around the wound, leading to excessive blood vessel proliferation, scar tissue formation, and tissue tightening during scar formation. During the fibrotic remodeling phase, TFs and MFs gradually undergo apoptosis, and the ECM forms a dense scar through the dehydration of collagen cross-links induced by selective degradation (30), which blocks the functional filtering bleb and disrupts aqueous humor drainage.

**Figure 1:**
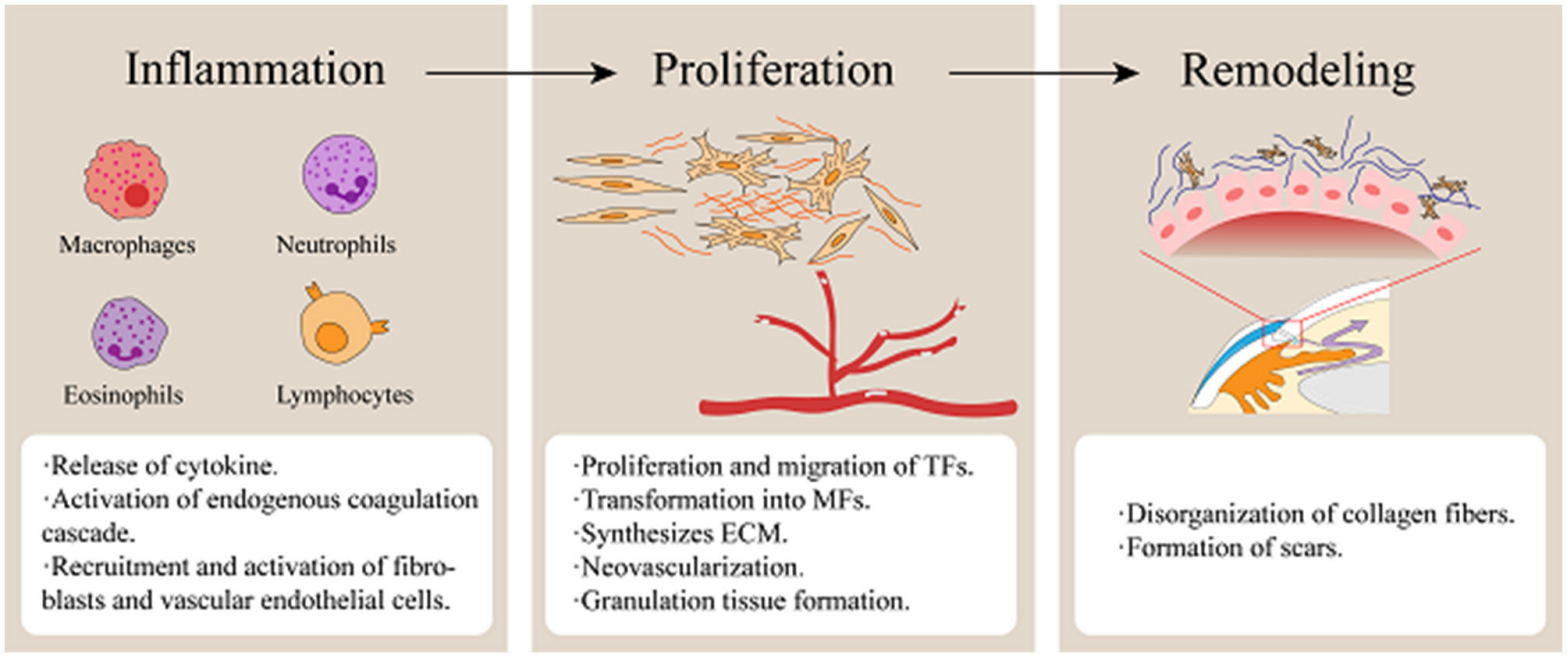
Stages of wound healing following glaucoma filtration surgery. (Fibrosis of filtering blebs following GFS is predominantly mediated by the proliferation, migration, and contractile activity of TFs. Surgical trauma triggers vascular disruption, releasing platelets and blood components that initiate coagulation cascades. Necrotic tissue debris, coagulative processes, and microbial infiltration collectively induce inflammatory activation. Inflammatory cells infiltrate the wound site to phagocytize cellular debris and pathogens while secreting growth factors and cytokines, including TGF-β, VEGF, PDGF, and IL-6. During the proliferative phase, neovascularization occurs while TFs are activated and transdifferentiate into MFs expressing α-SMA. Concurrently, ECM synthesis establishes a granulation tissue scaffold, facilitating wound contraction and repair. Ultimately, ECM remodeling occurs, culminating in the maturation of granulation tissue into dense fibrotic scar tissue).

In summary, fibrosis is a common pathological outcome of trauma and inflammatory responses, and excessive fibrosis leads to scar formation (Figure 1).

## 3 Current status of fibrosis of filtering bleb medication after GFS

To mitigate the fibrosis of the filtering bleb after GFS, glucocorticoids (31), and antimetabolites are commonly used in clinical practice. Corticosteroids are able to modulate fibroblast recruitment and inhibit their activity by alleviating the inflammatory response and reducing the release of inflammatory mediators. This supports their role in regulating the fibrotic process. However, glucocorticoids may increase the risk of postoperative infection (32). The antimetabolites mitomycin C (MMC) and 5-Fluorouracil (5-FU) are now commonly used clinical antifibrotic drugs. MMC, a broad-spectrum antitumor antibiotic isolated from Streptomyces, is a DNA cross-linking alkylating agent. MMC inhibits cellular DNA synthesis and replication, reduces cell proliferation, induces apoptosis in target TFs, and partially mitigates the fibrotic process (33). Compared with 5-FU, MMC is more effective and long-lasting in inhibiting the proliferation of fibroblasts, and it is considered the gold standard for mitigating postoperative fibrosis (34, 35). However, both MMC and 5-FU are associated with side effects such as thin-walled cystic blebs, late bleb leakage, bleb-related infections, chronic hypotony, hypotony maculopathy, and corneal epithelial toxicity (36–38).

Emerging experimental studies have revealed that immunosuppressive agents such as rapamycin augment cellular autophagic activity and, thus inhibit tissue fibrosis (39, 40). In *in vitro* experiments, bevacizumab has been shown to inhibit fibroblast proliferation by reducing new blood vessel formation and collagen deposition. It also suppress scar formation progression in animal models (41). Another study (42) demonstrated that the combination of MMC and bevacizumab enhances the success rate of GFS compared to MMC alone. Postoperative follow-up further revealed improved maintenance of filtering bleb morphology in patients. Intravitreal injection of ranibizumab has been shown to reduce angiogenesis and maintain postoperative filtering bleb morphology (43). Although anti-vascular endothelial growth factor agents exhibit anti-fibrotic effects, their use in this context remains controversial and requires further investigation (44). Rosiglitazone blocks the p38 signaling pathway. This inhibition suppresses TGF-β1-induced proliferation and differentiation of TFs and prolongs functional bleb survival (45). A novel protein, the S58 aptamer, targeting TGF-β receptor II, suppresses fibroblast transdifferentiation into myofibroblasts mediated by TGF-β2 (46). Comparative studies with mitomycin C (MMC) demonstrated a significant reduction in myofibroblast numbers in the S58 aptamer-treated group (47). However, the free S58 aptamer is susceptible to nuclease degradation and requires nanocarrier-based delivery systems (e.g., exosomes) (48). IP-10 peptide is a small-molecule cytokine that inhibits fibroblast migration, angiogenesis, and collagen deposition by binding to the CXCR3 receptor. It blocks VEGF-induced angiogenesis and promotes the regression of neovascularization (49). Studies have shown that IP-10p-treated filtering blebs exhibit reduced collagen deposition, decreased cell density, and inhibited scar formation (50). The vascular density of filtering blebs in the IP-10p group was also lower than that in the MMC-treated group. Additionally, the IP-10p combined with MMC group demonstrated reduced conjunctival damage compared to MMC alone. Animal experimental studies on Rho kinase (51) and matrix metalloproteinases (52) have shown antifibrotic efficacy. All these agents exhibit certain anti-fibrotic potential in filtering blebs and represent viable alternatives. However, their efficacy and safety in human glaucoma patients still require further clinical testing.

In recent years, active ingredients of traditional Chinese medicine have shown unique potential in inhibiting postoperative fibrosis in glaucoma due to their multi-target regulatory properties. Studies have found that homoharringtonine, an antimetabolic agent, acts as an anti-fibroblast proliferative agent by inhibiting DNA synthesis (53). It has been shown that Hansenulae mitigate fibrosis progression by inhibiting fibroblast proliferation through apoptosis and down-regulating filtration bleb fibrosis (54). Quercetin is a flavonoid compound. *In vitro* experiments revealed that it inhibits postoperative glaucoma fibrosis by inhibiting collagen synthesis and cell proliferation (55, 56). Although significant progress has been made in previous studies, herbal monomers still face challenges in antifibrosis research. Current research is still at the stage of *in vitro* and animal experiments, with a lack of dynamic simulation systems capable of replicating physiological aqueous microenvironments. Clarifying the mechanisms of herbal monomers in filtering bleb fibrosis and developing optimized delivery methods with precise dosages will establish a robust foundation for clinical trials, thereby enhancing the evidence base for clinical translation. Identification of herbal components with high efficacy, low toxicity, and high bioavailability is critical for postoperative antifibrotic studies in glaucoma.

SIN is a purified alkaloid from the traditional Chinese medicine *Sinomenium acutum* (57). SIN has been shown to reduce the formation of scar tissue in organs and tissues (58), including mitigating fibrosis in the lungs, liver, kidneys, and other tissues (59–63). Because of the similarity in the pathological mechanisms of fibrotic diseases, SIN is hypothesized to exert antifibrotic effects after GFS. Although the antifibrotic effect of SIN has not been widely applied in the field of ophthalmic diseases, its extensive research background, low toxicity, and high production yield suggest potential value (64). Investigating the pharmacological properties of SIN will help to understand its antifibrotic mechanisms after GFS and provide new therapies for postoperative fibrosis management. As a promising alternative or adjuvant agent, SIN warrants further exploration in preclinical models and clinical trials to validate its translational potential.

## 4 SIN prevents and suppresses fibrosis of filtering bleb after GFS

### 4.1 Anti-inflammatory

As the initiating phase of tissue repair and scar formation, reducing the inflammatory response helps inhibit postoperative filtering bleb fibrosis. Studies have shown that SIN reduces the secretion of inflammatory factors like IL-6, GM-CSF, IL-12 p40, IL-1α, IL-1β, TNF-α and other inflammatory factors in the serum of mice, and demonstrates significant anti-inflammatory activity (65). SINO-WCJ-33, a SIN derivative, significantly reverses elevated serum levels of IL-2, IL-6, and TNF-α in mice and plays an important role in modulating inflammatory responses (66). In ophthalmology research, SIN eye drops given to mice with experimental dry eye showed that the SIN-treated group exhibited significantly reduced corneal expression of IL-1β and TNF-α compared to controls, along with increased tear production (67).

Damaged cells and pathogens are cleared when the inflammatory response initiates, and subsequently neutrophils, macrophages, and lymphocytes are recruited to the injury site, producing clots, platelet-derived mediators, cytokines, and other factors. These mediators in turn induce fibroblast migration and transdifferentiate fibroblasts into MFs. These changes cause protein deposition and the formation of dense scar tissue (68). Previous research has demonstrated that therapeutic strategies targeting the inflammatory response after GFS reduce the progression of filtering bleb fibrosis. SIN exhibits significant anti-inflammatory effects, inhibiting the infiltration of inflammatory cells and the production of various cytokines, thereby reducing postoperative filtering bleb fibrosis (69, 70).

### 4.2 Inhibits fibroblast proliferation and promotes apoptosis

SIN significantly inhibits fibroblast proliferation and promotes apoptosis. By inhibiting TGF-β1/Smad3, PI3K/Akt, and NF-κB signaling pathways, SIN inhibits the migration and proliferation of fibroblasts and A549 cells. It prevents myofibroblast transdifferentiation and epithelial mesenchymal transition, resulting in reduced ECM protein expression (71). SIN has been shown to inhibit fibrosis progression by promoting collagen-I and collagen-III degradation through upregulation of ADAMTS-1 expression (72). After SIN treatment, fibroblasts exhibit reduced activity, decreased colony formation, and increased apoptosis rates. This suggests that SIN directly inhibits fibroblast proliferation while promoting apoptosis. These changes may be produced by upregulation of miR-23b-3p expression and downregulation of FGF9 expression in the miR-23b-3p/FGF9 axis (73). Similar to other fibrotic pathologies, involves excessive collagen and ECM deposition mediated by fibroblasts. Furthermore, another study reported the dose-dependent pro-apoptotic effect of SIN on human Tenon fibroblasts (74).

Western blot analysis showed that the TGF-β1 signaling pathway in human fibroblasts was significantly inhibited after SIN treatment. Meanwhile, the expression levels of Cyclin D1, Bcl-2, and MMP2 were significantly reduced. This study demonstrated that SIN suppresses fibroblast growth and migration, an effect mediated through inhibition of the TGF-β1 signaling pathway. These findings establish a mechanistic foundation for further exploration of SIN in anti-fibrotic therapy.

Fibroblasts are involved in fibrotic activities in the body and play an important role in promoting angiogenesis and granulation tissue formation. As wound healing proceeds to the cell proliferation stage, fibroblasts are recruited and activated to transdifferentiate into MFs, leading to increased collagen synthesis and deposition. This further drives fibrosis (75). In this stage, the key steps to mitigate fibrosis of the filtering bleb after GFS include: (1) inhibition of fibroblast migration and proliferation, and promotion of fibroblast apoptosis; and (2) suppression of MF transdifferentiation and induction of their apoptosis. A large body of evidence shows that SIN not only inhibits fibroblast migration, and proliferation but also promotes fibroblast apoptosis, reduces α-SMA expression, and enhances collagen degradation. SIN effectively inhibits the progression of fibrosis. TFs are one of the key effector cells in fibrosis of filtering bleb after GFS (76). SIN may suppress the fibrosis of the filtering bleb after GFS by modulating the activity of TFs and thereby maintaining functional filtering bleb to increase the success of the procedure.

### 4.3 Possible molecular mechanisms

The antifibrotic effect of SIN is mainly reflected in: (1) the anti-inflammatory effect of SIN attenuates the local inflammatory reaction after trauma or surgery, reduces inflammatory cell infiltration and cytokine release, and inhibits fibrosis at the injury site; and (2) SIN inhibits the activation, migration, and proliferation of fibroblasts, promotes their apoptosis, and suppresses their transdifferentiation into myofibroblasts. Based on its anti-inflammatory and antifibrotic pharmacological effects, evidence suggests that SIN helps maintain the function of the filtering bleb after GFS and prevent fibrosis. At present, studies on the molecular mechanism of SIN's antifibrotic effect on filtering bleb have mainly focused on the TGF-β/Smad, PI3K/AKT and NF-κB signaling pathways.

#### 4.3.1 TGF-β/Smad

TGF-β/Smad is a major pathway leading to scar formation, closely associated with ECM synthesis and fibroblast transdifferentiation (77, 78). Glaucoma patients exhibit elevated TGF-β concentrations in the aqueous humor and trabecular meshwork, suggesting that targeting TGF-β signaling represents a key therapeutic strategy for preventing postoperative fibrosis (79, 80). As a multifunctional dimeric polypeptide growth factor, TGF-β has been shown to promote proliferation, migration, and myofibroblast transdifferentiation of various target cells, and enhances the production of fibrosis-related proteins (81). The Smad protein family acts as TGF-β downstream intracellular effectors (82), with Smad2, Smad3, Smad4, and Smad7 being essential signaling components. Upon activation, TGF-β binds to the type II receptor (TβRII), which phosphorylates and activates the type I receptor (TβRI) kinase. This initiates the Smad-dependent signaling pathway, leading to phosphorylation and activation of downstream effectors such as Smad2 and Smad3, which mediate various biological effects (83–85). It was found that SIN-treated human fibroblasts not only exhibited cell cycle arrest but also underwent apoptosis. As the concentration of SIN increased, the number of apoptotic cells rose, accompanied by inhibition of TGF-β1 signaling. This was further evidenced by a decrease in the expression of Cyclin D1, a key regulator of the G1-to-S phase transition, and Bcl-2, an anti-apoptotic protein. In addition, the expression of MMP-2, which is associated with ECM remodeling and fibroblast migration, was also reduced. These findings indicate that SIN inhibits fibroblast proliferation and migration by regulating the TGF-β1/Smad signaling pathway (74). On the one hand, SIN reduces TGF-β release, inhibits its binding to cell surface receptors, and decreases ECM synthesis. On the other hand, it inhibits Smad protein phosphorylation, reduces their activation, and disrupts TGF-β signaling (86, 87).

#### 4.3.2 PI3K/AKT

The PI3K/AKT pathway consists of phosphatidylinositol-3-kinase (PI3K), protein kinase B (PKB), and its downstream molecules (88). PKB, also known as AKT, is a serine/threonine protein kinase and the principal downstream effector of the PI3K signaling pathway. Upon exogenous stimulation, PI3K is activated, leading to phosphorylation of AKT in cells and tissues. This activation induces a variety of biological effects, including the regulation of cell metabolism, growth, proliferation, and apoptosis. It also modulates oxidative stress, inflammatory responses, and energy homeostasis through multiple downstream targets (89). SIN attenuates renal fibrosis by modulating the PI3K-AKT pathway and affecting autophagy levels through BMSC-exo carrying miR-204-5p (90). Upon inhibition of PI3K activity, Akt phosphorylation and activation are reduced, which disrupts downstream signaling pathways, decreases cell proliferation and survival, and suppresses tissue fibrosis (91, 92).

#### 4.3.3 NF-κB

NF-κB is a family of transcription factors widely involved in regulation of cellular immunity, inflammation, proliferation, and apoptosis, primarily modulating inflammatory responses and cell survival (93, 94). As one of the classical inflammatory signaling pathways, NF-κB induces the transcription of various pro-inflammatory cytokines, promotes ECM synthesis, and contributes to fibrosis (95). SIN inhibits the nuclear translocation of NF-κB p65 subunit andthe DNA-binding activity of NF-κB in synoviocytes, which might be one of anti-inflammatory mechanisms (96). SIN increases A2A receptor and suppresses NF-κB pathway activation via the α7 nicotinic acetylcholine receptor in adjuvant-induced arthritis rats (97, 98). The inflammatory response is a key contributor to fibrosis. Suppressing the expression of inflammatory cytokines helps slow fibrotic progression, and SIN's antifibrotic effect is likely mediated through inhibition of NF-κB signaling.

## 5 Discussion and conclusion

GFS remains the gold-standard intervention for refractory glaucoma, but postoperative fibrosis of the filtering bleb is a leading cause of surgical failure. The pathogenesis of filtering bleb fibrosis is multifactorial, involving inflammatory, fibroblast proliferation and transdifferentiation, ECM deposition, aberrant angiogenesis, and pro-fibrotic cytokine signaling. This process is originally part of postoperative injury healing, but excessive fibrosis may lead to filtering bleb dysfunction. Although current therapies show some efficacy, they are frequently associated with considerable toxicity and adverse effects. Furthermore, several emerging therapies remain at the experimental stage, and the development of safe and effective agents to prevent or attenuate filtering bleb fibrosis after GFS remains a significant challenge.

Fibrosis of the filtering bleb after GFS shares a similar pathological mechanism with other fibrotic diseases. Although no studies have reported the effect of SIN on filtering bleb fibrosis, analysis of fibrosis mechanisms and SIN's pharmacological properties suggests its therapeutic potential. SIN exerts anti-inflammatory effects by reducing inflammatory reactions, decreasing inflammatory cell infiltration and cytokine release, thereby inhibiting fibrosis at the injury site. SIN inhibits the activation, migration, and proliferation of fibroblasts while promoting their apoptosis. It suppresses the transdifferentiation of fibroblasts into myofibroblasts. It decreases the expression of α-SMA, reduces protein deposition, and promotes collagen degradation. SIN's antifibrotic effects on filtering bleb fibrosis after GFS may involve the TGF-β/Smad, PI3K/Akt, and NF-κB signaling pathways.

The strong histamine-releasing effects of SIN may induce skin edema, pruritus, and gastrointestinal reactions (99), suggesting the need to be vigilant for patients with asthma and a history of allergy during clinical application. The study showed (100) that the distribution pattern of SIN in organs after multiple administrations was similar to that of a single administration, and no drug residues were detected in any tissue after 1 week of drug withdrawal, suggesting no accumulation in the body. It is worth noting that SIN concentrations are highest in the liver, and lower in the heart. Histological observation suggests relatively obvious morphological changes in liver tissue. However, no significant abnormalities were detected in liver function tests (ALT, AST), renal function (BUN), or urinary sediment in rats following 6 weeks of continuous administration and 1 week of withdrawal. This indicates that high-dose SIN did not compromise hepatic or renal enzymatic profiles, despite causing subtle histological alterations in the liver. Prolonged oral administration of SIN may induce hepatotoxicity, nephrotoxicity, and cardiotoxicity. Regular monitoring is therefore recommended for patients with severe hepatic or cardiovascular diseases. Additionally, caution is advised when using SIN in patients undergoing systemic immunosuppressive therapy for autoimmune diseases, as well as in pregnant or breastfeeding women (101, 102).

The safety profile of Sinomenine in ophthalmic applications is currently under investigation, with topical ocular administration considered effective for mitigating the aforementioned risks. After treatment with 0.05 and 0.1% Sinomenine (SIN) eye drops administered four times daily, slit lamp examination and corneal staining revealed no significant ocular irritation or corneal damage in mouse dry eye models (67). In rabbit uveitis models, administration of 0.5% SIN solution or gel showed no significant ocular irritation. Histopathological examination further confirmed that neither corneal epithelial detachment nor stromal damage occurred after treatment with 0.5% SIN solution or gel (103). In another study (104), the use of 1% SIN eye drops (four times daily for 7 days) significantly inhibited inflammation, reduced neovascularization, and promoted epithelial repair, effectively treating acute phase damage to the cornea in alkali burns. No corneal opacity, iris inflammation, or persistent conjunctival hyperemia was observed during the trial. The corneal epithelium and stromal structures remained intact with no pathological damage. However, the study lasted only 7 days, so long-term observation is still needed to explore its effect on subsequent scar formation.

By analyzing the mechanism of fibrosis in filtering bleb formation, current therapies, and SIN's pharmacological effects, SIN shows potential to maintain filtering bleb function after GFS. This analysis offers novel perspectives for studying anti-fibrotic strategies targeting the filtering bleb. Further studies are required to evaluate SIN's efficacy and safety, with the aim of providing a better therapeutic option for glaucoma surgery patients.
